# Low-Dose Fluoxetine in Four Children with Autistic Spectrum Disorder Improves Self-Injurious Behavior, ADHD-Like Symptoms, and Irritability

**DOI:** 10.1155/2018/6278501

**Published:** 2018-05-30

**Authors:** Juan Pablo Lucchelli, Gilles Bertschy

**Affiliations:** ^1^Ville-Evrard Hospital, Neuilly-sur-Marne 93330, France; ^2^Laboratory of Clinical Psychopathology, E.A. 4050 University of Rennes 2, Rennes, France; ^3^Department of Psychiatry, Mental Health and Addictology, Strasbourg University Hospital, Strasbourg, F-67000, France; ^4^INSERM U1114, Strasbourg, F-67000, France; ^5^Translational Medicine Federation, University of Strasbourg, F-67000 Strasbourg, France

## Abstract

Autism Spectrum Disorder (ASD) is defined by the copresence of two core symptoms: alteration in social communication and repetitive behaviors and/or restricted interests. In ASD children and adults, irritability, self-injurious behavior (SIB), and Attention Deficit and Hyperactivity Disorders- (ADHD-) like symptoms are regularly observed. In these situations, pharmacological treatments are sometimes used. Selective Serotonin Reuptake Inhibitors- (SSRI-) based treatments have been the subject of several publications: case reports and controlled studies, both of which demonstrate efficacy on the symptoms mentioned above, even if no consensus has been reached concerning their usage. In this article four clinical cases of children diagnosed with ASD and who also present ADHD-like symptoms and/or SIB and/or other heteroaggressive behaviors or irritability and impulsivity treated with low doses of fluoxetine are presented.

## 1. Introduction

ASD is defined by the copresence of two core symptoms: alteration in social communication and repetitive behaviors and/or restricted interests [1]. The diagnosis can be made very early, usually after the age of 2. Regarding the pharmacological treatment of ASD, it only concerns some of the symptoms, such as self-injurious behavior (SIB), irritability, anxiety, and ADHD-like symptoms. Several studies show the effectiveness of antipsychotic drugs, especially ones of a second-generation [2]; some controlled studies show an improvement of some core symptoms through treatment with SSRIs [3]. In general, however, there is little consensus concerning the most efficient type of SSRI treatment, and meta-analyses show that there is no clinical benefit to the adult and child ASD population using SSRIs; some even report that SSRI treatment may be harmful [4].

The use of SSRIs in ASD children has been the subject of several case report studies [5, 6] as well as some randomized clinical trials (RCT) [3, 7–9]. Cook et al. [6] used fluoxetine in 23 children and adults with ASD and mental retardation. This study reported improvement in some symptoms as well as significant side effects (probably related to the use of high doses: 20 to 80 mg/day). In a cross-over trial, Hollander et al. [3] observed an improvement in the repetitive behavior of 39 children with ASD using an average final dose of 9.9 mg/day of fluoxetine. Leventhal et al. [8] published a placebo treatment followed by randomized cross-over trial in which 15 children with ASD were treated with fenfluramine. This type of SSRI treatment increases serotonin release at a higher dose. The study did not, however, show any clear benefits in the usage of this treatment, despite improvement in hyperactivity as well as in emotional and social responses. King et al. [10] used citalopram in the treatment of 149 ASD in children, demonstrating a general lack of efficacy of citalopram. Hollander et al. [9] and McDougle et al. [11] demonstrated the efficacy of SSRIs in adults with ASD: Hollander et al. used fluoxetine in 37 adult patients and found improvement in repetitive behaviors (dose: 20-80 mg/day). McDougle et al. trial used fluvoxamine and found an improvement in aggression and inappropriate responses in 30 adult patients (dose max. 300 mg/day).

In this article, the authors present four clinical cases of ASD-diagnosed children with ADHD-like symptoms and/or SIB and/or other heteroaggressive behaviors and/or irritability and impulsivity. Each was treated with low doses of fluoxetine, specified as follows: 2.5 mg/d (liquid formulation) in the morning for the first week, followed by a flexible titration schedule based on weight and tolerability. The Hollander et al. protocol [3] is reproduced here, in which children with ASD were given low doses of fluoxetine. Patients were assessed using the Clinical Global Impression Scales (CGI) [12] during the time of fluoxetine introduction and observation. None had tried an SSRI treatment before the reported trial.

## 2. Case 1

An 8-year-old girl (19 kg) had an ASD diagnosis according to the DSM-5 and ADI-R criteria based on information provided by parents. She also had significant mental retardation, with severe SIB (banging her head against objects and biting her hands), forcing her entourage to maintain a daily and permanent physical restraint. She spends most of her time in a day hospital. She received the following pharmacological treatment: risperidone 2 mg/d and cyamemazine 80 mg/d without modifications to her SIB and at the price of a major slowing down and a manifestation of a tendency toward blunting. The CGI severity of illness score was at five (markedly ill). We decreased and stopped risperidone and started valproic acid. After four weeks of valproic acid 400 mg/d in combination with cyamemazine (60 mg/day), SIBs did not improve. Then, we added fluoxetine 2.5 mg/d and increased it after one week to 5 mg/d and to 10 mg/d in the third week. After one week, the CGI improvement scale (CGI-I) was at two; after three weeks, it lowered to 1 (very much improved). We also observed a significant decrease in anxiety as well as the disappearance of SIB (disappearance of the behavior consisting of the banging and rubbing her head against objects). However, it should be noted that the entourage kept the bandages on her hands because she continued to bite them, even if she did it with less intensity than before. There were no side effects. After three months of fluoxetine, her clinical state remains stable.

## 3. Case 2

A 12-year-old boy (70 kg), with DSM-5 criteria for an ASD and ADI-R confirming this diagnosis, exhibited extreme irritability, violence, and impulsiveness as well as SIB (he had thrown seven television sets out of the window). The CGI severity illness scoring was at six (severely ill). In the day hospital where he spent most of his time, it was difficult for staff to manage his impulsivity and unpredictability. His treatment included risperidone 4 mg/d as well as loxapine 80 mg/d. Despite this pharmacological treatment, episodes of aggression and SIBs continued. This treatment induced a significant weight gain (8 kg in 5 months). Treatment with fluoxetine 2.5 mg/d was introduced and increased to 5 mg/d after one week and to 10 mg/d at the beginning of the third week. After one week, there was a CGI-I score of three, which decreased to two after two weeks of treatment and to one after three weeks. Such a positive clinical response allowed for a reduction in risperidone to 2 mg/d and in loxapine to 60 mg/d. The treatment was tolerated well by the patient, and he began to lose weight (4 kg). After two months of fluoxetine, his clinical state remains stable.

## 4. Case 3

A 6-year-old male child (30 kg) with DSM-5 criteria and ADI-R for an ASD exhibited problems of SIB and repetitive behaviors (washing his hands for more than 30 minutes at least two to three times per day), severe irritability, frequent crying, social withdrawal, and inappropriate speech. Treatment with risperidone 2 mg/d had improved irritability and partially the SIB, but it had also produced significant weight gain (four kg in three months). A decrease in the risperidone dosage seemed necessary. Treatment with fluoxetine 2.5 mg/d was begun, which quickly led to a reduction in inappropriate behavior (for example, impulsive crawling on the ground in the classroom). After one week, the CGI-I scoring was at two. The dosage was gradually increased to 5 mg/d the second week and to 7.5 mg/d the third week. The repetitive behaviors gradually subsided. After three weeks the CGI-I score was at one, and it remained stable for nine weeks. The risperidone dosage could be decreased to 0,5 mg/day and the patient's weight remained the same.

## 5. Case 4

A 12-year-old boy (62 kg) with DSM-5 and ADI-R criteria for a severe case of ASD, including severe ADHD-like symptoms, often required physical restraint and did not improve despite a long-term treatment of risperidone 3 mg/d as well as melatonin 4 mg at bedtime. The CGI severity illness scoring was at 6 (severely ill). The behavioral pattern included irritability, marked agitation, crying, severe hyperactivity, and other behaviors typical of this disorder. He was also anxious, rendering the situation at his day hospital where he spent most of his time all the more difficult. A prescription of fluoxetine 2.5 mg/d was initiated with an immediate and complete improvement of ADHD-like symptoms: CGI-I at one week of treatment was at a one, making this case the most remarkable of the four presented here. Treatment with fluoxetine was continued with a dosage increase up to 5 mg/d to allow for a decrease in the risperidone dose to 1 mg/d. CGI-I score remained stable at one for the duration of the nine weeks.

## 6. Discussion

These four cases suggest that treatment of SIB and ADHD-like symptoms as well as irritability in patients diagnosed with ASD can be improved by low doses of fluoxetine (mean dose: 8.75 mg/d; range: 5-10 mg). In all four cases, we observed a rapid improvement in hyperactivity, restlessness, and inadequate behaviors. The measured CGI-I scores were almost at two and three by the end of one week of treatment and even lowered to one, as demonstrated in Case 4.

It should also be noted that all of our patients were treated with antipsychotic drugs (risperidone), which had side effects: apart from weight gain, there was also some emotional and cognitive impairments (especially in cases 1 and 2). With the decrease in antidopaminergic drugs, children became more connected cognitively and emotionally. In Case 1, risperidone 2 mg/d not only did not improve SIB, but it also left the child in a state of emotional blunting. After the cessation of risperidone and the introduction of fluoxetine a few weeks later, the child stopped banging his head and started a kind of self-sensory activity, which consisted of touching and caressing her legs and thighs. One could even draw the hypothesis that she began to appropriate her body image and her interoceptive awareness by improving her interoceptive abilities which were blocked by the antipsychotic treatment [13]. For Case 2, an ASD child with major impulsivity and fluoxetine, combined with risperidone and loxapine, decreased his aggressive tendencies without producing side effects (insomnia), apart from desirable weight loss. Regarding Case 3, ASD with a compulsive component (hand washing in particular), the introduction of fluoxetine notably improved inadequate and compulsive behavior and anxiety. Regarding Case 4, a 50 kg child with nonverbal ASD with ADHD-like symptoms, the addition of 2.5 mg / d of fluoxetine produced the most dramatic improvement with almost immediate progress, evaluated one week after treatment with a CGI-I score of one. The most interesting aspect is the speed of the clinical response to fluoxetine: for instance, a rapid clinical development was observed in Case 4 when an improvement in ADHD-like symptoms (and anxiety) was seen within 48 hours of the initial dose (2,5 mg/day). To our knowledge, such rapid responses to low doses have not been emphasized in the literature. Also concerning Case 4, it could be possible that the improvement of ADHD-like symptoms is the result of decrease in anxiety. One might also specify the notion of “low dose”: given the weight of children, the ratio dose/weight is not insignificant and can be compared to 20 mg/day standard doses in adults for Case 1 and Case 3. However, it is important to note that in several studies the dose range was higher than the standard 20 mg dose in adults and in children [3, 6, 7]. It is also interesting to cite works concerning venlafaxine, a Noradrenalin-Serotonin Reuptake-Inhibitor which, at a low dose (18,75 mg/day), is rather a SSRI, demonstrating benefits in adolescents and adults with ASD.

Concerning the possible mechanisms of action, several perspectives could be discussed. They could be dependent upon some of the questions that our case reports left unanswered. First, one might ask if there is an onset action which is shorter than the antidepressant action which is considered to be linked to the serotonin reuptake inhibition. If yes, one might also ask if this is a sufficient argument for the consideration of another mechanism of action. This question could be addressed in connection with the next point. Secondly, one might pose the question as to whether or not fluoxetine has a specific role (would we obtain the same results with other SSRI?). As mentioned in the introduction, fluoxetine is the SSRI most examined in child, adolescent, and adult ASD-related behavioral problems. One large, open study with citalopram in children with ASD reported no benefits [10]. One study with fluvoxamine in adults with ASD showed positive results [11]. Should fluoxetine have a specific role, it could be interesting to remember that fluoxetine, unlike other SSRIs, increases norepinephrine and dopamine extracellular levels in prefrontal cortex of mice, probably through 5-HT2C receptors blockade [14] and that fluoxetine seems to be the only SSRI at risk of abuse [15]. Thirdly, one might ask if such a low-dose SSRI is indeed sufficient. If the answer is yes, would it be possible to consider alternative mechanisms? For instance, SSRIs seem to act as a selective brain steroidogenic stimulant at very low doses that are inactive on 5-HT reuptake [16].

In conclusion, in these case reports, we found that the prescription of fluoxetine, in addition to valproate and cyamemazine (Case 1) or to risperidone (Cases 2, 3 and 4), could be effective on severe behavioral symptoms associated with ASD in children. It is important to inform child psychiatrists about this therapeutic possibility even if it would be difficult to predict the rate of responders on the basis of this cases and the literature. The role of comedication remains unanswered as none of our cases was on fluoxetine monotherapy.
